# *PbGA20ox2* Regulates Fruit Set and Induces Parthenocarpy by Enhancing GA_4_ Content

**DOI:** 10.3389/fpls.2020.00113

**Published:** 2020-02-18

**Authors:** Huibin Wang, Ting Wu, Jianlong Liu, Liu Cong, Yanfei Zhu, Rui Zhai, Chengquan Yang, Zhigang Wang, Fengwang Ma, Lingfei Xu

**Affiliations:** College of Horticulture, Northwest A&F University, Yangling, China

**Keywords:** gibberellin, gibberellin 20-oxidase, parthenocarpy, fruit set, fruit development, pear

## Abstract

Fruit set and development occur following successful fertilization. Parthenocarpy, a valuable trait in some self-incompatible species, produces seedless fruit without fertilization. Gibberellin (GA) is a crucial hormone in fruit-set regulation and development. While investigating the development of parthenocarpy in pear (*Pyrus bretschneideri* Rehd.), we determined that GA 20-oxidases (GA20ox) may play key roles in seedless pear fruit development. Sequence analysis revealed three *PbGA20ox* genes: *PbGA20ox1, PbGA20ox2*, and *PbGA20ox3*. We analyzed the expression patterns of candidate genes and found that *PbGA20ox2* levels significantly changed in pollinated fruits. Tissue-specific expression assays revealed that *PbGA20ox2* is highly expressed in young fruit and leaves. Subcellular localization assays showed it was located in the cytoplasm, nucleus, and plasma membrane. Overexpressed *PbGA20ox2* tomato plants were taller and had longer hypocotyls and internodes, and the emasculated flowers produced parthenocarpic fruit. In pear, the transient overexpression of *PbGA20ox2* promoted fruit development and delayed the drop of nonpollinated fruit. Furthermore, the fruit of *PbGA20ox2-*overexpressing tomato and transient *PbGA20ox2-*overexpressing pear had increased GA_4_ (but not GA_3_ and GA_1_) contents. This result provided evidence that *PbGA20ox2* was necessary for GA_4_-dependent pear fruit development. Our study revealed that *PbGA20ox2* altered the GA biosynthetic pathway and enhanced GA_4_ synthesis, thereby promoting fruit set and parthenocarpic fruit development.

## Introduction

Fruit set, a key developmental process that occurs following successful fertilization in higher plants, is the first step in fruit development (Joldersma and Liu, 2018). Fruit set and development represent the commitment of an angiosperm plant to the development of mature fruit (Joldersma and Liu, 2018). In many fruiting plants, pollination and fertilization are necessary for fruit set and development, with these events leading to the development of seeds that promote cell division and fruit development in a synchronized manner (McAfee et al., 2013).

‘Dangshansu’ (*Pyrus bretschneideri* Rehd.), a pear cultivar with fleshy fruits, is highly studied because of its considerable economic importance. In most fruiting species, fruit set and development are triggered by pollination and fertilization. However, many fruit species, including pear, exhibit natural self-incompatibility. Parthenocarpy, which induces seedless fruit, is independent of pollination and fertilization and is a valuable trait for some self-incompatible species.

Plant hormones play key roles in parthenocarpic fruit growth. Parthenocarpy can be induced by the exogenous application of plant hormones, such as gibberellins (GAs), auxins, and melatonin (Cong et al., 2018; Liu et al., 2018a; Liu et al., 2018b), as well as the regulation of endogenous hormone contents (Mesejo et al., 2010; Ding et al., 2013; Sugiyama et al., 2014; Niu et al., 2015). Application of GAs can be used for this purpose in many horticultural plants, such as apple (Watanabe et al., 2008), loquat (Aslmoshtaghi and Shahsavar, 2013) and pear (Liu et al., 2018b). Genes related to GA biosynthetic or signaling pathways may also have functions in parthenocarpic fruit development. In tomato, the silencing of the *GA 2-oxidase* (*GA2ox*) gene, which encodes a deactivating enzyme in the GA biosynthetic pathway, induces parthenocarpic fruit and inhibits lateral branching (Martínez-Bello et al., 2015). The silencing of the *DELLA* gene, which negatively regulates GA signaling, induces facultative parthenocarpy in tomato fruit (Martí et al., 2007). In pea, the overexpression of *GA3ox,* which converts inactive GA to active GA, induces parthenocarpic fruit (Reinecke et al., 2013).

In ‘Dangshansu’ pear, exogenous application of GA_4+7_ (Gibberellin A4 and A7), but not GA_3_, can induce parthenocarpy (Liu et al., 2018b). GA plays important roles in regulating diverse developmental processes (Hedden and Sponsel, 2015), such as shoot elongation, germination, flowering, fruit set, and growth (Hedden and Kamiya, 1997; Olszewski et al., 2002). The GA metabolic pathways have previously been described (Singh et al., 2010; Hedden and Thomas, 2012). GAs are synthesized from *trans*-geranylgeranyl pyrophosphate and metabolized to GA_12_ and/or GA_53_. The two precursors are converted to active GA_4_ and GA_1_ by GA20ox and GA3ox (García-Hurtado et al., 2012). GA20ox plays a key role in this pathway and is involved in the successive oxidation of C-20, leading to its removal and the formation of C_19_-GAs (GA_9_ and GA_20_) (Rieu et al., 2008a). GA_4_ and GA_1_ are synthesized from GA_9_ and GA_20_, respectively. In brief, two parallel pathways exist that include consecutively acting GA20ox: non-13-hydroxylation and early-13-hydroxylation pathways (**Supplementary Figure S1**). *GA20ox* has been cloned and characterized in many plants. For example, five *GA20ox* genes are present in Arabidopsis, six in grape and at least four in tomato (Serrani et al., 2008; Rieu et al., 2008b; Giacomelli et al., 2013). In these species, *GA20ox* is involved in diverse developmental processes, including vegetative and reproductive growth.

Here, we reported that *PbGA20ox2* regulated fruit set and development by increasing the contents of specific active GAs during an early stage of fruit development in pear. Using quantitative real-time PCR (qRT-PCR), tissue-specific expression analyses and subcellular location assays, we investigated the expression patterns of *PbGA20ox2*. Furthermore, we investigated the functions of *PbGA20ox2* in fruit set and development using a stable genetic overexpression assay in tomato and a transient overexpression assay in pear.

## Materials and Methods

### Plant Material, Growth Conditions and Treatments

Experiments were carried out in a pear orchard located in Meixian, Shaanxi Province, China (34.29°N, 107.76°E; 514 m above sea level). The average annual precipitation at this location was 574.6 mm, and the average annual temperature was 12.7°C. During anthesis, the average temperature was 12°C.

‘Dangshansu’ pear, which was grafted onto *P. betulifolia* Bge. rootstock, was used for the study. Two days before anthesis, all treated and control plants were bagged to prohibit pollination. Healthy and uniform plants were subjected to three treatments: (i) application of water to unpollinated flowers, serving as the nonpollination treatment (Control); (ii) spraying of a solution of 50 mg L^−1^ GA_4+7_ (GA) [the concentration previously determined by Liu et al. (2018b)] on unpollinated ‘Dangshansu' flowers at anthesis followed by immediately bagged, and (iii) hand pollination (P), performed at the same time. The three treatments were carried out on three trees, and three branches were selected on each three. Three branches from each treatment were used as three replicates, and each treatment contained about 150 flowers. The whole fruits were collected at 0, 3, 9, and 14 days after treatment (DAT), and each sample consisted of 60 fruits at every time point. Ovules were separated from 30 fruits which were collected at 0, 3, 9 DAT and used for qRT-PCR. Pear is a kind of pseudocarp, and the hypanthiums develop into fruit. The information of pear floral organ was shown in **Figure S2**, and the sample of pear labeled as fruit included hypanthiums, ovules, and ovaries in this study. For a tissue-specific expression assay, total RNA was isolated from the whole fruits (including hypanthiums, ovules, and ovaries), pedicels, sepals, styles, young leaves, and young stems at 6 DAA (days after anthesis) respectively. Each sample was frozen in liquid nitrogen and stored at −80°C for further analyses.

Tomato (*Solanum lycopersicum* L. ‘Micro-Tom') was used in the transgenic experiments. Plants were placed in pots (120 × 100 mm) containing a mixture of peat: vermiculite (1:1 v/v) in a growth chamber at 25°C under 16-h/8-h light/dark conditions and irrigated daily with Hoagland's nutrient solution (García-Hurtado et al., 2012).

### Isolation of *PbGA20ox* Family Genes

*GA20ox* genes were identified from the pear-genome coding DNA sequence (CDS) database (Wu et al., 2013) (http://www.ncbi.nlm.nih.gov/genome/?term = pyrus). Hidden Markov Model profiles of *GA20-ox* family gene conserved domains (PF14226 and PF03171) were obtained from the Pfam database (Pan et al., 2017) and used as queries for searching against the pear genome database. To confirm the existence of a corresponding highly similar EST sequence for each predicted gene, sequences of candidate genes were queried against the pear genome EST database (Zhai et al., 2016) using BLASTN. Alignment of amino acid sequences of the candidate genes was performed using ClustalW (Thompson et al., 1997).

### Transcriptome Analysis

Transcriptome data generated in our previous study (Liu et al., 2018b) were used to analyze fruit development-related genes. The gene expression data were obtained from samples of unpollinated fruits, unpollinated fruits subjected to GA_4+7_ treatment, and pollinated fruits collected at three different stages: 3, 9, and 14 days after anthesis.

Clean reads were mapped using Bowtie 1.12 to generate read alignments for each sample (Langmead et al., 2009). Gene expression levels were calculated as fragments per kilobase of exon model per million mapped reads (FPKM) (Wapinski et al., 2013). RNA-seq data of unpollinated fruits were used as the controls. In order to avoid missing important genes, we did not set the minimum value of FPKM. The pairwise comparison of FPKM values includes nine combinations, P *vs* UP, P *vs* GA, GA *vs* UP at 3 DAA, 9 DAA, and 14 DAA, respectively. The results were used as references to screen candidate genes. Three independent biological replications were sequenced and analyzed.

### Phylogenetic Analysis

Sequences of designated pear *GA20ox* genes were downloaded from the pear genome database (Wu et al., 2013) (http://www.ncbi.nlm.nih.gov/genome/?term = pyrus) and aligned with *GA20ox* genes from Arabidopsis, apple, tomato, citrus, rice, and grape using ClustalW. The phylogenetic analysis was performed using the minimum-evolution method under the JTT model in MEGA 5.0. Information on the analyzed genes was listed in **Supplementary Table S5**.

### Cloning of the *PbGA20ox2* Gene

The CDS of *PbGA20ox2* (LOC103960493) was cloned from cDNA of ‘Dangshansu' pear using primer sets (**Supplementary Table S3**) designed from the sequence data in the pear genome database (http://www.ncbi.nlm.nih.gov/genome/?term = pyrus). The methods of RNA extraction and RNA reverse-transcription are described below. The PCR amplification was carried out using the following protocol: 40 cycles at 98°C for 10 s, 55°C for 15 s and 72°C for 10 s. The primers are based on CDS and described in **Table S3**.

### Expression Analysis by qRT-PCR

Total RNA was extracted using a RNAprep Pure Plant kit (Tiangen, Beijing, China). RNA concentration and quality were assessed by UV spectrophotometry and on a 0.8% agar ethidium bromide-stained gel, respectively. Next, 1 μg of total RNA was reverse-transcribed to cDNA using a PrimeScript RT reagent kit with gDNA Eraser (Takara, Dalian, China). qRT-PCR amplifications were performed on an ABI instrument (Thermo Fisher Scientific, Massachusetts, USA) using a SYBR Premix Ex *Taq* kit (Takara) and selected gene primers, which were designed with Primer Premier 5.0 software (Liu et al., 2018b). qRT-PCR amplifications were carried out using the following protocol: initial incubation at 95°C for 30 s, followed by 40 cycles of 95°C for 5 s and 60°C for 30 s. All reactions were performed with three biological repeats. Data were collected by AB StepOne Plus software (Thermo Fisher Scientific, Massachusetts, USA) and we used the ΔΔCT algorithm to analyze the results (Livak and Schmittgen, 2001). The primers for actin genes of pear and tomato, *PbGA20ox1, PbGA20ox2*, and *PbGA20ox3* were described in **Table S3**.

### Subcellular Localization of *PbGA20ox2*

The full-length CDS of *GA20ox2* was cloned into a pCambia2300–green fluorescent protein (GFP) to form a translation fusion with the N-terminus of the GFP. The vector was kindly provided by Professor Xu Yan, Northwest A&F University. *Agrobacterium tumefaciens* strain EHA105, containing either a pCambia 2300 vector with 35S::GFP or the GA20ox2—35S::GA20ox2-GFP, was grown at 28°C in Luria–Bertani medium containing 50 mg L^−1^ kanamycin and 25 mg L^−1^ rifampicin. After 24 h, the *Agrobacterium* cells were harvested and resuspended in infiltration buffer [10 mM MgCl_2_, 10 mM MES (pH 5.6) and 150 mM acetosyringone] to a final OD_600_ of 0.8. The resuspended cells were shaken for 4 h at room temperature and then subjected to infiltration by a syringe. The methods of infection were based on Hellens et al. (2005). The leaves were incubated in the dark at 22°C for 12 h and then placed in a growth chamber (25°C, 16-h/8-h day/night) for 4 to 5 days. Fluorescence Microscopic (BX51 + PD72 + IX71, OLYMPUS, Japan) imaging system was used to observe the anatomical images. Excitation and emission wavelengths were 498 nm and 516 nm respectively.

### Production of Transgenic Tomato Plants

The complete CDS of *PbGA20ox2* was fused to the CaMV 35S promoter of a pCambia 1301 binary vector (**Supplementary Figure S3**). The construct 35S::GA20ox2 was transformed into tomato using *Agrobacterium tumefaciens* strain EHA105 as described in García-Hurtado et al. (2012). Briefly, the tomato first leaf sections (from 20-day-old seedlings grown under sterile conditions) were cultured for 2 days in the dark in Petri dishes containing solidified preculture (PC) medium [MS salts supplemented with vitamins, 3% (w/v) sucrose, 100 mg/L myo-inositol, 4 mg/L indole acetic acid (IAA), 4 mg/L kinetin, and 0.8% (w/v) agar]. Then they were immersed in bacterial suspensions (OD_600nm_ = 0.4) containing 200 µM acetosyringone for 10 min. Blotted explants were cultured in the dark for 2 days in solidified PC medium with 200 µM acetosyringone, washed in washing medium [MS salts, 2% (w/v) sucrose, 100 mg/L myo-inositol, and 500 mg/L cefotaxime], and cultured for 2 days in the dark in solidified PC medium. Then the explants were transferred to PC medium containing 1 mg/L zeatin, 300 mg/L cefotaxime, and 100 mg/L kanamycin. Explants developing resistant calli produced shoots, which were excised and placed on rooting medium [MS salts, 2% (w/v) sucrose, 100 mg/L myo-inositol, 1 mg/L thiamine, 0.1 mg/L IAA, and 0.8% (w/v) agar]. Rooted explants were cultured in pots containing vermiculite, watered with Hoagland's solution, and conditioned in a growth chamber before transferring to the greenhouse.

RNA was extracted from young leaves, and used for qRT-PCR to identify the positive transgenic lines. The specific primers: 5′-CAATGGCACTCCATTAGCCC-3′ (sense) and 5′-TTCACTGTTCACCACTGCCCT-3′ (antisense) were used. The method of qRT-PCR was described as above. Progeny from the transgenic plants were obtained by selfing under controlled conditions. According to the phenotypes, we designed three experiments of self-cross. Then we obtained homozygous lines in the third generation, which were used for the study and replicated each transgenic line in tissue culture.

### Tomato Germination and Growth Conditions

Tomato seeds from transgenic lines and wild-type (WT) plants were imbibed in 50°C water for 4–5 h. The seeds were then bagged in wet gauze, placed in 90-mm-diameter plastic Petri dishes and sprayed with double-deionized water every 3 h at 25°C in the dark. After germination for 48 h, the seedlings were transferred to pots (120 × 100 mm) containing a mixture of peat: vermiculite (1:1, v/v) and cultured in a growth chamber (25°C, relative air humidity of 70%–80%, 16-h/8-h day/night photoperiod and photon flux of 115 μ mol m^–2^ s^–1^). Seedlings were irrigated daily with Hoagland's solution, and the natural light was supplemented with Osram lamps (Powerstar HQI-BT, 400W) to provide 16 h of daylight (Martínez-Bello et al., 2015).

### Transgenic Tomato Phenotypes

For each line, we recorded the following phenotypes: (i) hypocotyl length (mean length from bottom of the stem to cotyledon); (ii) height to first inflorescence (mean length from soil surface to first inflorescence); (iii) internode length (average of three internode lengths between first to fourth leaves); (iv) stem diameter (stem diameter of third internode); (v) leaves to first inflorescence; (vi) root length (measured 5 days after seed germination in the growth chamber); and (vii) days to anthesis (time to first open flower).

### Parthenocarpic Capacity of Transgenic Tomato

We analyzed the parthenocarpic capacity of transgenic tomato in two independent assays, each involving four independent lines (WT, 10-1, -6 and -8). Two or three trusses and three to four emasculated flowers were left on at least nine plants per line in two independent experiments. Flower emasculation was carried out 2 days before anthesis to prevent self-pollination, and all non-selected flowers were removed. The percentage of developed fruits and their weights were determined at maturity.

### Transient Overexpression Assay in Pear Inflorescences

The complete CDS of *PbGA20ox2* was fused to the CaMV 35S promoter of a pCambia 1301 binary vector (35S::GA20ox2) (**Supplementary Figure S3**). *Agrobacterium* cell growth and resuspension methods were the same as those used in the subcellular localization assay described above. Flowers at 3 DAA and at same position (the center flowers of the inflorescence) were used. Plant infiltration was performed as follows: a syringe with the needle removed was fixed on the inflorescence. 3–4 flowers on each inflorescence for the infiltration and the unpollinated inflorescence was dipped in the *Agrobacterium* infiltration buffer. **Figure S4** is a simplified diagram of the experiment. During dipping, negative pressure was maintained for 10 min by pull syringe piston. We then removed the syringe, and the inflorescence was bagged to prohibit pollination. The negative controls were infiltrated with *Agrobacterium* containing empty pCambia 1301 vectors (**Supplementary Figure S3**). At 5 DAT, We randomly selected fruits to analyze *PbGA20ox2* expression levels in the control and the two lines overexpressing *PbGA20ox2* by qRT-PCR. Starting at 11 DAT, we measured fruit size, including fruit length and diameter, every 3 days until fruit drop. The measuring of the fruit size was carried out in three biological repeats, each with 7 fruits at 11 DAT and 14 DAT and the sample randomly selected fruit in the middle of inflorescence.

For GUS staining, plant materials were stained with 5-bromo-4-chloro-3-indolyl glucuronide at 37°C for 12 h as described in Fillatti et al. (1987).

### Phytohormone Analysis

Gibberellins levels were determined using high-performance liquid chromatography–tandem mass spectrometry (AB SCIEX TripleTOF 5600+, Darmstadt, IN, USA). Approximately 0.5 g of fruits was ground in liquid nitrogen and extracted using solvent A (1 L 80% methanol containing 0.5 g citric acid and 0.2 g butylated hydroxytoluene). The sample was extracted with 4 ml of solvent A, and the extract was shaken at 4°C for 12 h. After centrifuging the sample at 10,000 ×*g* and 4°C for 15 min, the supernatant was collected. Next, 2 ml of solvent A was added to the sample, which was shaken again at 4°C for 1 h. The sample was centrifuged at 10,000 ×*g* at 4°C for 15 min, and the supernatant was collected. This step was repeated. All supernatant aliquots were mixed and dried under nitrogen gas, dissolved in 250 µl methanol and passed through a 0.22-µm filter membrane. The high-performance liquid chromatography analysis was performed using an ACQUITY UPLC HSS T3 (1.8 μm, Waters, USA) column (2.1 × 100 mm). The mobile phase solvent was the same as in Balcke et al. (2012), and the injection volume was 2 µl. The mass spectrometry conditions were as follows: a spray voltage of 4,500 V, and air curtain, nebulizer, and auxiliary gas pressures of 15, 65, and 70 psi, respectively. The atomizing temperature was 400°C. Each sample consisted of three replicates from independent experiments.

### Statistical Analyses

Data were subjected to analysis of variance and tested for significant (*P < 0.05) treatment differences using Duncan's test. Results were presented as the means ± standard deviation (SD) of three replicate samples.

## Results

### Isolation and Identification of *GA20ox* Genes in Pear

**Figure 1:**
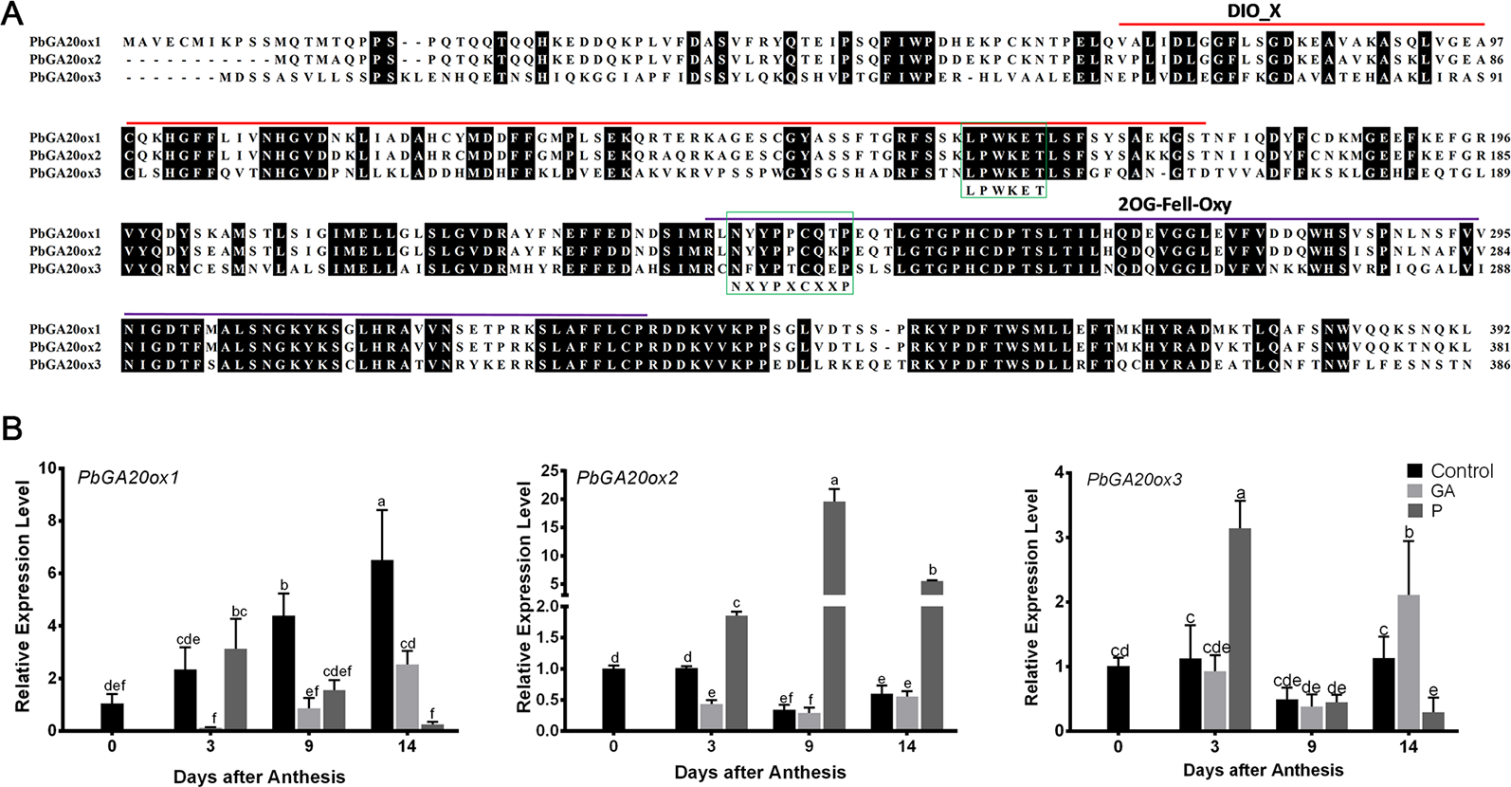
Alignment of amino acid sequences of *PbGA20ox*s and their expression models after different treatments. **(A)** Alignment of the three amino acid sequences. DIO_X and 2OG-Fell-Oxy domains are marked with bold lines, and identical residues are shaded in black. The proposed conserved sequences ‘NXYPXCXXP’ and ‘LPWKET’ are indicated by green boxes. **(B)** Relative expression levels of *PbGA20ox* genes. Relative transcript quantities were determined by qRT-PCR. The expression level of each gene in fruits under control conditions at 0 DAT was normalized to 1.0. The results shown are means ± SD (*n* = 3). Different letters between bars indicate significant differences at *P* < 0.05 (Duncan's range test). Control, nonpollinated; GA, gibberellin treatment; HP, hand pollination.

In our previous study, we found that GA_4+7_ induced seedless pear fruit (Liu et al., 2018b); to investigate the mechanism by which GA induces parthenocarpy in pear, we analyzed the RNA-seq data, which showed that pollination induced the expression of gibberellin biosynthesis pathway related genes, such as*, PbGA3ox, Ent-Kaurene Synthase*, *Ent-Kaurene Oxidase,* and *PbGA20ox* genes, and the expression of *PbGA20ox2* was increased dramatically. The expression of *PbGA2ox2*, which transfers active GAs to inactive GAs, was downregulated by pollination at 9 DAA (**Supplementary Table S1**).These results indicated that pollination promoted GAs biosynthesis. GA20ox, as a rate-limiting enzyme, plays a key role in the GA synthesis pathway, so we inferred that it may be related to fruit development. On the basis of conserved domains of the *GA20ox* subfamily, we identified three *PbGA20ox* genes in pear, *PbGA20ox1* (LOC103942611), *PbGA20ox2* (LOC103960493), and *PbGA20ox3* (LOC103957121). Amino acid sequence alignment uncovered two conserved domains, DIOX_N and 2OG-Fell_Oxy, and indicated that the sequence ‘LPWKET’ was highly conserved in all *PbGA20ox* genes (**Figure 1A**). Phylogenetic analysis revealed that *PbGA20ox1* and *PbGA20ox2* are most closely related to *MdGA20ox1c* and *MdGA20ox1a*, while *PbGA20ox3* is similar to *MdGA20ox5* and *VvGA20ox1, 3, 5* (**Supplementary Figure S5**). To explore *PbGA20ox* functions, we quantitatively assessed the expression profiles of the three *PbGA20ox* genes (**Figure 1B**). Hand pollination increased the expression of *PbGA20ox2* dramatically, whereas *PbGA20ox2* expression was reduced in GA-treated nonpollinated fruits compared with nonpollinated ones (**Figure 1B**). *PbGA20ox1* and *PbGA20ox3* gene expression levels were reduced in pollinated fruits at 9 DAA; their expression levels after pollination were lower than in fruit treated with exogenous GA at 14 DAA, although the expression of *PbGA20ox3* increased at 3 DAA (**Figure 1B**). We also quantitatively assessed the three genes in ovules. The result showed *PbGA20ox2* increased at 3 DAA and 9 DAA in the pollinated ovules, but the *PbGA20ox1* and *3* didn't increase in pollinated ovules (**Supplementary Figure S6**). On the basis of these results, *PbGA20ox2* was considered to be a candidate gene for further study.

### Tissue-Specific Expression Analysis of *PbGA20ox2*

**Figure 2:**
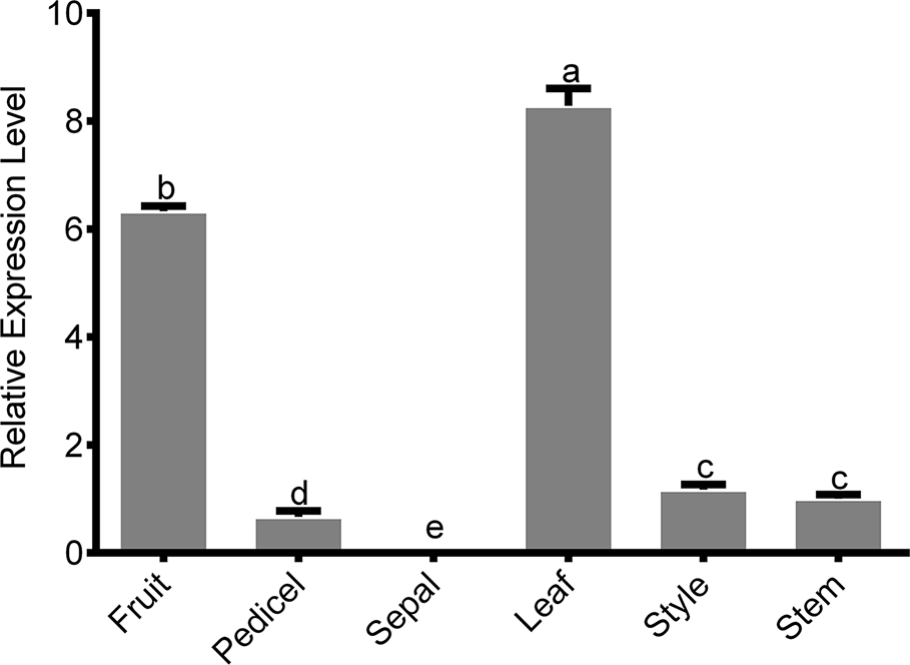
Tissue-specific expression of *PbGA20ox2*. A qRT-PCR analysis was used to determine the relative expression level of PbGA20ox2 in various tissues and organs. Values shown are means ± SD (*n* = 3). Significant differences (*P* < 0.05) among different tissues as determined by Duncan's test are indicated using different lowercase letters.

The biosynthesis of active GAs is a complex, multistep process, and GA biosynthetic genes are differentially expressed among different tissues, developmental stages, and cell types (Binenbaum et al., 2018). We analyzed the expression of *PbGA20ox2* in different tissues using qRT-PCR. *PbGA20ox2* was highly transcribed in fruits, which contain (hypanthium, ovary, and ovules), and leaves, with comparatively lower expression levels in pedicels, styles, and stems but no expression in sepals (**Figure 2**). *PbGA20ox1* and *PbGA20ox3* were highly transcribed in fruits (**Supplementary Figure S7**).

### Subcellular Localization of *PbGA20ox2*

**Figure 3:**
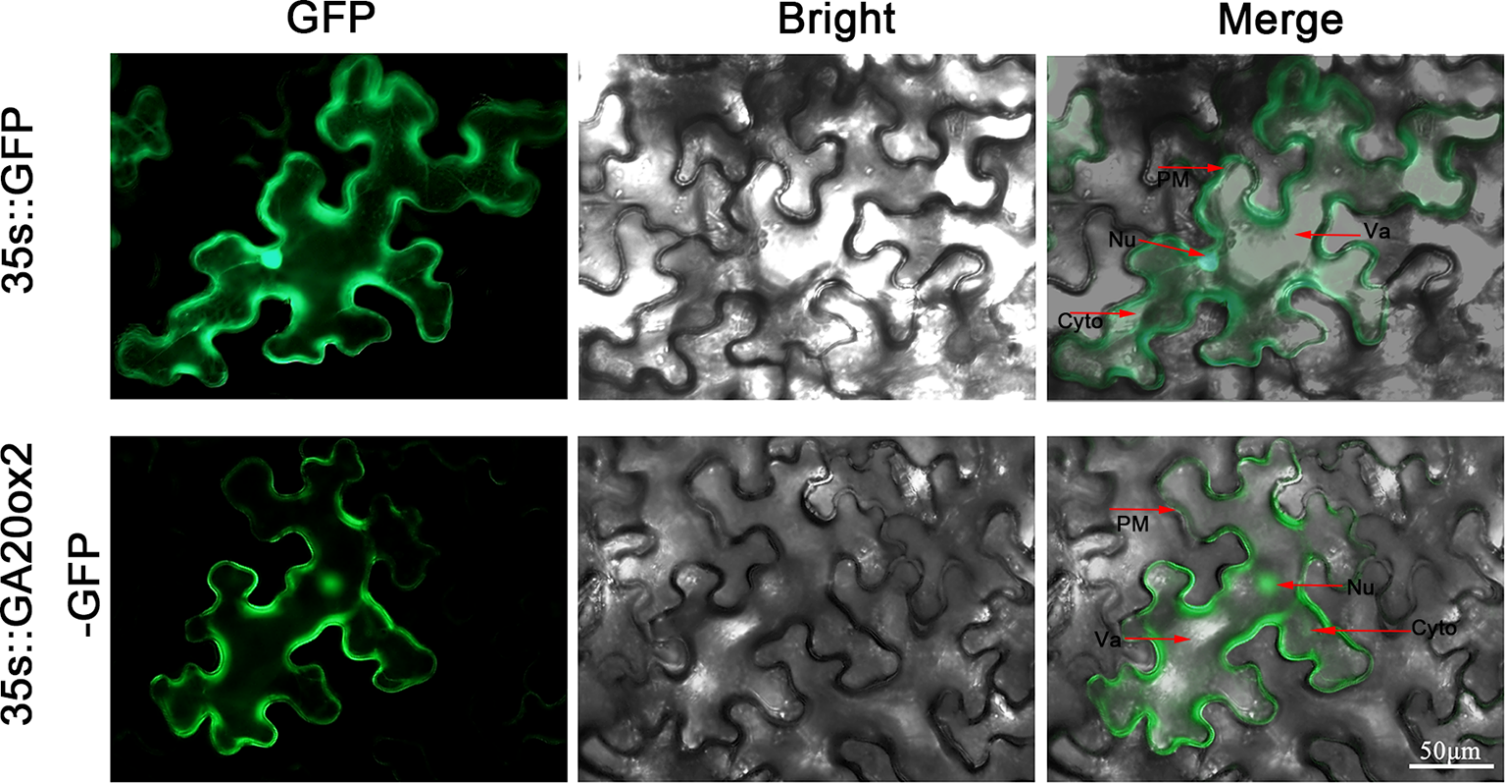
Subcellular localization of *PbGA20ox2* in tobacco leaves. The fusion protein (PbGA20ox2-GFP) and positive control (GFP) were independently transiently expressed in tobacco leaves. *PbGA20ox2* was detected in the cytoplasm, nucleus, and plasma membrane. PM, plasma membrane; Nu, nucleus; Cyto, cytoplasm; Va, vacule.

To determine the precise location of *PbGA20ox2* expression, we fused the coding sequence of *PbGA20ox2* to a GFP protein under the control of the CaMV 35S promoter (35S::PbGA20ox2-GFP), with the empty vector (EV, 35S::GFP) used as a control. We independently transiently expressed these constructs in tobacco leaves. Four days after transformation, the GFP fluorescence signal was examined using a fluorescence microscope. Merged bright and green fluorescence images are presented and the results suggested that PbGA20ox2 is localized in the cytoplasm, nucleus, and plasma membrane (**Figure 3**).

### Production of Parthenocarpic Fruit in *PbGA20ox2*-Overexpressing Tomato

**Figure 4:**
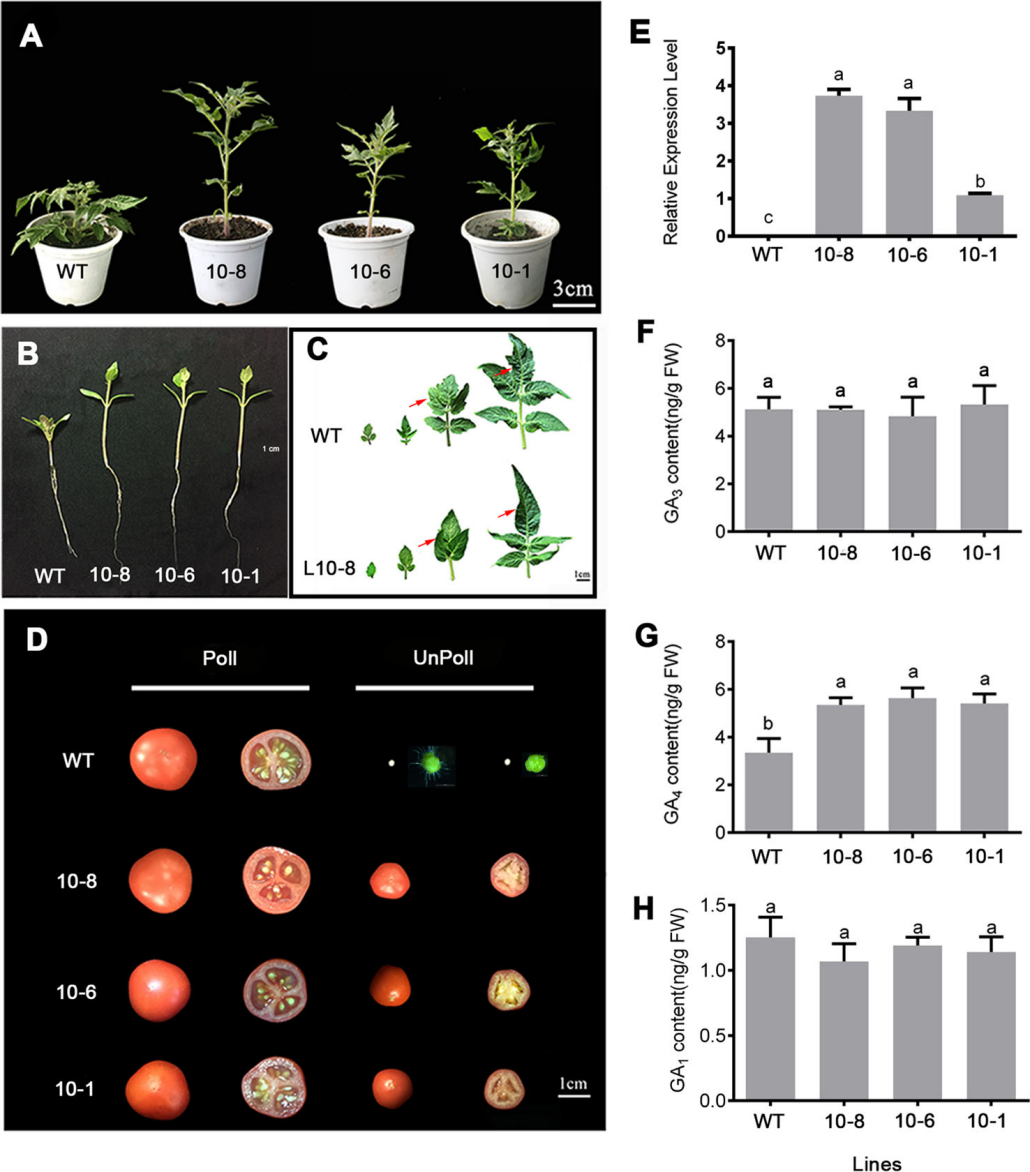
Phenotypes of wild-type (WT) and PbGA20ox-OE lines (10-8, -6, and -1) of tomato. **(A)** Plant before flowering. **(B)** Roots of a representative 10-day-old seeding. **(C)** Leaves at different positions on the plant (first to fourth, left to right). The difference points were marked with a red arrow. **(D)** Fruits of WT and GA20ox-OE lines. **(E)** Expression levels of *PbGA20ox2* in young leaves of WT and transgenic lines as assessed by qRT-PCR. Line 10-1 was used as control. Ten-day-old fruits were used for phytohormone analysis. **(F)** GA_3_ content. **(G)** GA_4_ content. **(H)** GA_1_ content. Data are means ± SD (*n* = 3). Different letters between bars indicate significant differences at *P* < 0.05 (Duncan's range test). Poll, pollinated; Unpoll, nonpollinated.

To investigate the function of *PbGA20ox2* in fruit set and development, we obtained transgenic tomato lines overexpressing the *PbGA20ox2* gene (*GA20ox2*-OE). Compared with the controls, plants of the transgenic lines were taller and had longer hypocotyls and roots, thinner stems and longer internodes (**Figures 4A, B**; **Supplementary Table S2**). The leaves of transgenic lines had smooth edges, whereas the WT had the characteristic serrated borders (**Figure 4C**). In addition, the flowering time and number of leaves before the first inflorescence were also changed in transgenic tomato lines (**Supplementary Table S2**). We obtained 10 positive lines, 3 of which (10-1, -6 and -8) were selected for analysis. A qRT-PCR assay verified the expression of *PbGA20ox2* in young leaves of three lines (**Figure 4E**), while a phytohormone analysis revealed significant increases in the content of GA_4_, but not of GA_3_ and GA_1_, in the fruitlets of three lines (**Figures 4F–H**).

To determine the function of *PbGA20ox2*, we analyzed fruit development in transgenic tomato lines and analyzed the parthenocarpic capacity of WT and *PbGA20ox2-*OE lines (10-1, -6 and -8) in two independent experiments. In total, 10.0–15.5% of nonpollinated fruits developed and produced parthenocarpic fruit in transgenic lines (**Figure 4D**, **Table 1**). In contrast, the rate of parthenocarpic fruit was 0 in WT lines unpollinated (**Figure 4D**, **Table 1**). In regard to fruit set and weight, the pollinated fruits of transgenic and control lines were not significantly different: the fruit-set rate was 100% with pollination, and the weights per fruit were ~4–5 g in the two types of lines (**Table 1**). In addition, the average weight of a parthenocarpic fruit was 0.8–0.9 g (**Table 1**). No mature fruits were generated from nonpollinated fruits of WT plants (**Figure 4D**). Consequently, *PbGA20ox2* altered vegetative growth and induced parthenocarpic fruit development in tomato.

**Table 1:** Fruit-set capacity of wild-type (WT) and *PbGA20ox2*-OE lines (10-8, -6, and -1) in two independent experiments.

	Lines	Pollinated		Non-pollinated	
		Fruit set (%)	Fruit weight (g fruit^−1^)	Fruit set (%)	Fruit weight (g fruit^−1^)
Experiment I	WT	100	4.2 ± 0.2	0(0/45)	–
	10-8	100	4.2 ± 0.8	12.9(8/62)	0.9 ± 0.05
	10-6	100	4.6 ± 0.1	10.7(11/103)	0.8 ± 0.07
	10-1	100	4.5 ± 0.2	10.2(8/78)	0.8 ± 0.06
Experiment II	WT	–	–	0(0/30)	–
	10-8	–	–	15.5(9/53)	0.8 ± 0.04
	10-6	–	–	13.1(11/84)	0.8 ± 0.06
	10-1	–	–	11.4(8/70)	0.9 ± 0.07

The pollinated fruit data came from 30 flowers in five plants. The nonpollinated fruit data were generated from emasculated flowers, hence resulting in different numbers of trusses per plant. Each truss had 3–4 treated flowers from 9 to 13 plants. Values in parentheses indicate the number of ovaries set over the total number of nonpollinated fruits. The results are means ± SD.

### Delayed Fruit Drop in *PbGA20ox2*-Overexpressing Pear

**Figure 5:**
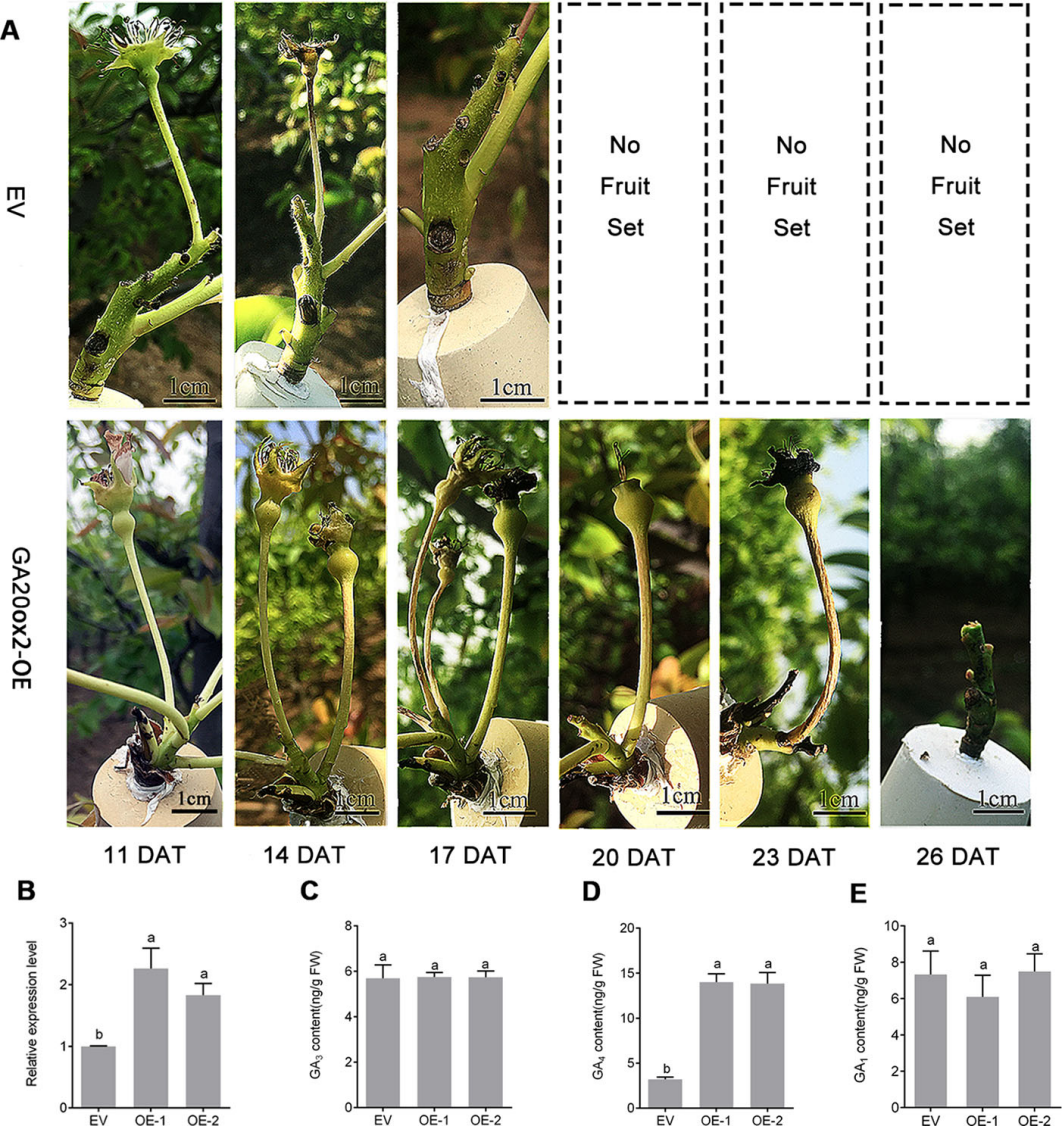
Transient overexpression assay of *PbGA20ox2* in ‘Dangshansu' pear fruits. **(A)** Images of inflorescence development on different days after the transient overexpression of *PbGA20ox2* in ‘Dangshansu' pear. **(B)** Relative expression levels of *PbGA20ox2* in control (empty vector, EV) and two independent overexpressed lines. The expression level of the gene in fruits transformed with the EV was normalized to 1.0. **(C–E)** GA_3,_ GA_4_ and GA_1_ contents in pear fruits at 5 DAT. OE-1 and OE-2 represent two biological repeats with overexpressing *PbGA20ox2*. Results are means ± SD (*n =* 3). Different letters between bars indicate significant differences at *P* < 0.05 (Duncan's range test). DAT, days after treatment.

To further determine the functions of *PbGA20ox2* in fruit development, we overexpressed the construct 35S::*PbGA20ox2* in pear inflorescences (*GA20ox*-OE), with the EV used as the control. Fruit infection was validated by monitoring GUS signals (**Supplementary Figure S8**). Fruit retention rate percentages were respectively 68.3 and 93.5% in control and *GA20ox2*-OE at 11 DAT. In addition, 53.9% of control fruit had dropped at 14 DAT, but 79.8% of fruits were set in *GA20ox*-OE. At 17 DAT, all control fruits had dropped, while *GA20ox*-OE fruit dropped at 26 DAT (**Figure 5A**, **Table 2**). We analyzed the expression of *PbGA20ox2* at 5 DAT by qRT-PCR, which revealed an increased expression of *PbGA20ox2* under the control of the CaMV 35S promoter in fruits compared with the control (infiltrated EV) (**Figure 5B**). Overexpression of *PbGA20ox2* also increased the expression of *GA 3-oxidase* and genes associated with cell division and expansion in fruits (**Supplementary Figure S9**). We also measured the fruit size of *PbGA20ox2*-transiently overexpressed and control (EV) fruits and found significant increases in *PbGA20ox2*-overexpressed fruit compared with the control (**Supplementary Table S4**). Furthermore, we determined that a significant change in GA_4_ content, but not GA_1_ and GA_3_ content, occurred at 5 DAT (**Figures 5C–E**). To conclude, *PbGA20ox2* promoted fruit development and delayed unpollinated fruit drop in pear by enhancing GA_4_ content.

**Table 2:** Fruit retention rate of ‘Dangshansu’ pear after the transient overexpression of *PbGA20ox2*.

Treatments	11 DAT(%)	14 DAT(%)	17 DAT(%)	20 DAT(%)	23 DAT(%)	26 DAT(%)
EV	68.3 ± 9.2 (45/66)b	46.1 ± 4.2 (31/66)c	0 (0/66)f	0 (0/66)f	0 (0/66)f	0 (0/66)f
GA20ox-OE	93.5 ± 3.0 (66/70)a	79.8 ± 6.9 (56/70)b	27.4 ± 2.5 (19/70)d	9.7 ± 0.5 (7/70)e	5.4 ± 1.1 (4/70)e	0 (0/70)f

Fruit retention rate values are means of three locations (± SD). Data are means of 60–70 flowers. Values in parentheses represent the number of retentive fruit over the total number of fruits. Values followed by a different letter are statistically significant at *P* < 0.05 (Duncan's range test). EV, empty vector; GA20ox-OE, *GA20ox* overexpression; DAT, days after treatment.

## Discussion

Fruit set in higher plants requires pollination. In fruiting plants, ovarian cell division is temporarily stopped at anthesis until pollination and fertilization occur (Reig et al., 2018). During this process, GA biosynthetic pathways are active in ovules (Dorcey et al., 2009). In this study, we isolated *PbGA20ox* genes, which may be related to fruit development. On the basis of conserved domains of the *GA20ox* family, we identified three *PbGA20ox* genes (**Figure 1A**). As assessed by a qRT-PCR assay, *PbGA20ox2* was significantly increased in pollinated fruits and was therefore selected as a candidate gene (**Figure 1B**). The expression profile of *PbGA20ox2* was similar between fruit and ovules, but the expression profile of *PbGA20ox3* showed a difference between fruits and ovules, so we inferred that the different expression patterns of *PbGA20ox3* may be related to its low expression in pear at the early stage (**Figure 1B**; **Supplementary Figure S6**). We next analyzed the location of *PbGA20ox2* expression. The biosynthesis of active GAs is a complex, multistep process, and GA biosynthetic genes are differentially expressed among different tissues, developmental stages, and cell types (Binenbaum et al., 2018). In rice flowers, GA biosynthetic genes are extremely highly expressed in the tapetum cells of anthers (Hedden and Phillips, 2000). In *Arabidopsis thaliana*, *GA20ox1* is expressed in the receptacle, and *GA20ox3* is expressed in the leaves, roots, hypocotyls, and internodes, with the five *AtGA20ox* genes mainly expressed in different locations (Plackett et al., 2012). In our study, *PbGA20ox2* was mainly expressed in leaves and fruits, with relatively lower expression detected in pedicels, styles, and stems and none in sepals (**Figure 2**). We have confirmed *PbGA20ox2* was related with fruit development, and we inferred that *PbGA20ox2* may also play important roles in the leaf. Additionally, our subcellular localization assay revealed that *PbGA20ox2* was localized in the nucleus, cytosol, and plasma membrane (**Figure 3**).

To determine the function of *PbGA20ox2* in fruit development, we overexpressed *PbGA20ox2* in tomato. We detected significant alterations in the architecture and vegetative growth of transgenic tomato (**Figure 4**, **Supplementary Table S2**). The similar alterations have been observed in diverse plants overexpressing *GA20ox* genes of different species, such as aspen (Eriksson et al., 2000), tobacco (Vidal et al., 2001), and switchgrass (Do et al., 2016). The morphological changes in these species were consistent with the higher expression of *GA20ox* and bioactive GA levels.

In transgenic tomato, emasculated flowers produced parthenocarpic fruit (**Figure 4D**), but the percentage of seedless fruit set was lower, and fruit size was smaller than that of pollinated fruit (**Table 1**). The limited parthenocarpic capacity of transgenic tomato was probably due to the relatively lower increase in GA_4_ in fruits compared with that resulting from the exogenous application of GAs to tomato (García-Hurtado et al., 2012). Furthermore, we overexpressed *PbGA20ox2* in pear inflorescences through a transient expression assay. Fruit drop was delayed approximately 9 days in GA20ox-OE compared with the control (**Figure 5A**, **Table 2**), and GA_4_ contents increased in transgenic tomato and pear fruits (**Figures 4G** and **5D**). Following the expression of *PbGA20ox2,* the *PbGA3ox* gene was expressed at a higher level in transgenic pear fruits relative to nontransformed ones (**Supplementary Figure S9**). In our case, however, the different pear fruit development patterns between GA20ox-OE and control may have been due to the greater viability of transgenic fruits and therefore was an indirect effect of *PbGA20ox2* overexpression. During pollination and fertilization, GA synthesis is activated in ovules, and fruit development begins during this process (Dorcey et al., 2009). In previous studies, transgenic tomato in which *CsGA20ox* was overexpressed (García-Hurtado et al., 2012) or *SlGA2ox* was silenced (Martínez-Bello et al., 2015) was found to induce parthenocarpy, and the active GA content increased. GA also negatively regulates the formation of the abscission layer to prevent fruit drop (Olsson and Butenko, 2018). Therefore, we confirmed that the overexpression of *PbGA20ox2* induced parthenocarpic fruit and delayed fruit drop by increasing GA_4_ content.

We also analyzed the reasons for fruit drop in the transient overexpression assay. Pear undergoes fruit set and development after GA treatment (Liu et al., 2018b). In the transient overexpression assay, all fruit dropped by 26 DAT (**Table 2**). After overexpression of *PbGA20ox2*, the increased GA_4_ content of young transgenic pear fruit did not lead to a fruit set rate as high as that of pollinated fruit (Liu et al., 2018b). We thus infer that the transient overexpression of *PbGA20ox2* cannot increase GA_4_ content to the same extent as that obtained by GA treatment (Liu et al., 2018b), and this observation is correlated with the fruit drop and low parthenocarpic fruit-set rate of transgenic tomato, which was same with García-Hurtado et al. (2012). The GUS staining assay also suggested that the low efficiency of agro-infiltration is directly related to the expression of *PbGA20ox2* and the content GA_4_ in the fruit, which is ultimately and indirectly related to fruit drop. Furthermore, the negative pressure conditions during infiltration in the transient expression assay caused physical injuries to the inflorescence, which might be correlated with fruit drop.

GA_1,_ GA_3,_ GA_4_ and GA_7_ are the most common active GAs in higher plants (Ding et al., 2013). In different species, the major active GAs may be different (Dorcey et al., 2009). If a plant has two functional GA forms, then different activity levels may exist, and GA abundance or mode of activity may vary between organs and across developmental stages. In tomato, the main pathway of GA biosynthesis is the early-13-hydroxylation pathway, which can produce GA_1_ and GA_3_ (García-Hurtado et al., 2012). In Arabidopsis, GA_4_ is the major bioactive form and promotes vegetative growth, floral initiation, and maturity (Eriksson et al., 2006). In rice, GA_4_ is the main active form associated with reproductive growth, while GA_1_ is the major bioactive form associated with vegetative growth (Kobayashi et al., 1989). In grapes, GA_4_ content increases during later fruit developmental stages (Giacomelli et al., 2013). Exogenous application of GA_4_ promotes vegetative and reproductive growth in tomato (García-Hurtado et al., 2012) and cucumber (Qian et al., 2018). Furthermore, the affinity of GA_4_ to bind GA INSENSITIVE DWARF1 (GID1), a GA signal receptor, is approximately 20 times greater than that of GA_3_ in rice (Ueguchitanaka et al., 2007). We predicted that GA_3_ and GA_4_ may have different functional stages in ‘Dangshansu' pear, an idea that needs further study. GA_1_ and GA_4_ represent two parallel GA synthetic pathways. In transgenic tomato fruit and pear fruits, GA_4_ increased while GA_1_ and GA_3_ remained unchanged (**Figures 4** and **5**). We thus infer that GA_4_ accumulation in early developmental stage is important for fruit set and development in ‘Dangshansu' pear and that *PbGA20ox2* plays key roles in these processes. Combined with the results of tissue specific experiments, we inferred that PbGA20ox2 may mainly catalyze GA_12_ in fruit at an early stage of fruit development. The involvement of *PbGA20ox2* in the functional differences between GA_4_ and GA_3_ in pear fruit development also requires further study.

## Conclusions

In this study, we demonstrated that *PbGA20ox2* plays key roles in fruit set and development. Overexpression of *PbGA20ox2* in tomato altered the vegetative phenotype and promoted the production of parthenocarpic fruit. In transient overexpression assays, in addition, the overexpression of *PbGA20ox2* delayed pear fruit drop. An analysis of endogenous GAs levels in transgenic tomato and pear revealed that *PbGA20ox2* promoted fruit set and development by increasing the accumulation of GA_4_ (but not GA_3_) at an early stage of fruit development.

## Data Availability Statement

All datasets generated for this study are included in the article/**Supplementary Material**.

## Author Contributions

HW, JL, RZ, and ZW designed the experiments. HW, TW, JL, LC, and YZ performed the experiments. HW analyzed the data. HW, RZ, CY, ZW, FM, and LX wrote and revised the manuscript. All authors have participated in this research and approved the final manuscript.

## Funding

This work was supported by the China Agriculture Research System (CARS-28-45) and Weinan Experimental Station foundation of Northwest A&F University.

## Conflict of Interest

The authors declare that this research was conducted in the absence of any commercial or financial relationships that could be construed as a potential conflict of interest.
