# Supplementary material for: PbGA20ox2 Regulates Fruit Set and Induces Parthenocarpy by Enhancing GA4 Content
Source: Front Plant Sci. 2020 Feb 18;11:113. doi: 10.3389/fpls.2020.00113 (PMC7039935; doi:10.3389/fpls.2020.00113)
Supplement: Supplementary file 1 [file DataSheet_1.docx]

**Supplementary Table S2** Phenotype of wild type and *PbGA20ox2* transgenic tomato lines (L10-8, -6, -1).

| Lines |  | **Hypocotyl length(mm)** | **Height to first inflorescence(cm)** | **Internode length**^a^**(cm)** | **Stem diameter**^b^**(mm)** | **Leaves to first inflorescence(n)** | **Root length**^c^**(mm)** | **Days to anthesis**^e^**(n)** |
| --- | --- | --- | --- | --- | --- | --- | --- | --- |
| WT |  | 21.9±1.4 | 7.9±0.4 | 8.5±06 | 4.3±0.2 | 7.9±0.3 | 73.4±1.3 | 29.8±0.2 |
| **L8** |  | 38.7±1.8 | 13.8±0.6 | 28.2±0.5 | 3.6±0.1 | 9.1±0.1 | 100.0±1.6 | 36.3±0.7 |
| **L6** |  | 41.8±0.9 | 11.7±0.4 | 24.3±0.5 | 3.4±0.1 | 9.0±0.3 | 88.5±1.7 | 38.6±0.4 |
| **L1** |  | 41.9±1.2 | 12.5±0.5 | 21.1±1.2 | 3.5±0.1 | 8.8±0.3 | 94.0±1.4 | 34.4±0.6 |

Data are means of 10 plants, ±SE. The compare of two groups was based on the tissue at the similar position and age on the plant.

^a^The data mean the average of three internode length from first to fourth leaf.

^b^Thedatamean the third internode stem diameter.

^c^Root length were measured 5 days after the seeds germinated and grew in the growth chamber (25℃).

^e^Days to anthesis were determined by the time of the first open flower.

**Supplementary Table S3 The list of primers**

| Assay | Primers | Forward(5’-3’) |  | Reverse(5’-3’) |
| --- | --- | --- | --- | --- |
| qRT-PCR | *PbGA20ox1* | GGTGACAAAGAAGCCGTCG |  | CATGTAGCAGTGAGCGTCCG |
| qRT-PCR | *PbGA20ox2* | CAATGGCACTCCATTAGCCC |  | TTCACTGTTCACCACTGCCCT |
| qRT-PCR | *PbGA20ox3* | TCTCTCGGAACCGGACCTC |  | GTCGAACCGAATGCCACTTC |
| qRT-PCR | *PbGA3ox-1* | GTGGTCAATGTTGGTGATCTCATT |  | ATGGATAGACGATGTTGGGTGC |
| qRT-PCR | *PbCyclin D3-1* | TCTTGGGAATTCTGGGCATCA |  | ACGCTTGTTGGATTGTTTGCC |
| qRT-PCR | *PbCyclin A2-2* | TTGAACACCACTGGTTGCCT |  | TCGAAAGAGTGAACCACCCG |
| qRT-PCR | *PbExpension A4* | ATCTGCATGGTGTGTTGGCA |  | ACCACATGCTCCTCCCATTG |
| qRT-PCR | *PbExpension A10* | CTGAAAATGGCTCTTTTCGGGA |  | AAGGTTGCATGAGCATTGGAC |
| qRT-PCR | *Slactin* | GACAGGCGTTCAGGTAAGG |  | CCAATGGAGGGTATTCAGC |
| qRT-PCR | *Pbactin* | TGAGTCACACTGTGCCAATCTATG |  | TGGTGAACATGTACCCTCTTTCAG |
| Tansgenic Tomato | *p*Cambia1301-*PbGA20ox2* | GGGACTCTTGACCATGCAAACCATGGCACAAC |  | TTCGAGCTGGTCACCTCACAGTTTCTGGTTGGTTTTC |
| Transient overexpression  Subcellular localization | *p*Cambia1301-*PbGA20ox2*  *p*Cambia2300-*PbGA20ox2* | GGGACTCTTGACCATGCAAACCATGGCACAAC  AGCTCGGTACCCGGGGATCCATGCAAACCATGGCACAAC |  | TTCGAGCTGGTCACCTCACAGTTTCTGGTTGGTTTTC  CTTGCTCACCATGGTGTCGACCAGTTTCTGGTTGGTTTTC |

*Slactin*：the *actin* gene of tomato (*Solanum lycopersicum* L.)

**Supplementary Table S4** Fruit size of the ovary after infiltrated with the 35S::GA20ox2 construct in comparison with the empty vector.

| **Treatments** |  |  | **11DAT** | **14DAT** | **17DAT** | **20DAT** | **23DAT** |
| --- | --- | --- | --- | --- | --- | --- | --- |
| **EV** | Fruit length/mm |  | 3.65 ± 0.26 h | 2.65 ± 0.13 j | - | - | - |
|  | Diameter/mm |  | 3.14 ± 0.37 i | 3.12 ± 0.13 i | - | - | - |
| **GA20ox-OE** | Fruit length /mm |  | 5.58 ± 0.15 f | 6.82 ± 0.39 cd | 7.88 ± 0.12 a | 7.27 ± 0.37 bc | 7.20 ± 0.18 bc |
|  | Diameter /mm |  | 4.84 ± 0.10 g | 6.23 ± 0.77 e | 6.49 ± 0.18 de | 7.52 ± 0.25 ab | 6.90 ± 0.18 cd |

The data of fruit length mean the longitudinal diameter of fruit. The data of diameter mean the maximum diameter which is perpendicular to the fruit length. Values followed by a different letter are statistically significant at *P* < 0.05 (Duncan’s range test). EV, empty vector; GA20ox-OE, *GA20ox2* over-expression; DAT, day after treatment; -, no fruit.

**Supplementary Table S5** Detailed annotations of *GA20ox* genes in seven species.

| Species | Name | Accession no | Reference |
| --- | --- | --- | --- |
| *Pyrus bretschneideri* | *PbGA20ox1* | XP_009351069.1 | - |
|  | *PbGA20ox2* | XP_009371252.2 | - |
|  | *PbGA20ox3* | XP_009367490.1 | - |
| *Malus domestica* | *MdGA20ox1a* | MDP0000248981 | Guitton, et al., 2012 |
|  | *MdGA20ox1b* | MDP0000136940 | Guitton, et al., 2012 |
|  | *MdGA20ox1c* | MDP0000280240 | Guitton, et al., 2012 |
|  | *MdGA20ox1d* | MDP0000128715 | Guitton, et al., 2012 |
|  | *MdGA20ox2* | MDP0000142765 | Guitton, et al., 2012 |
|  | *MdGA20ox4* | MDP0000508761 | Guitton, et al., 2012 |
|  | *MdGA20ox5* | MDP0000136905 | Guitton, et al., 2012 |
| *Vitis vinifera* | *VvGA20ox1* | GSVIVT01008782001 | He, et al. 2019 |
|  | *VvGA20ox2* | GSVIVT01018453001 | He, et al. 2019 |
|  | *VvGA20ox3* | GSVIVT01019696001 | He, et al. 2019 |
|  | *VvGA20ox4* | GSVIVT01026453001 | He, et al. 2019 |
|  | *VvGA20ox5* | GSVIVT01026466001 | He, et al. 2019 |
|  | *VvGA20ox6* | GSVIVT01027572001 | He, et al. 2019 |
|  | *VvGA20ox7* | GSVIVT01031837001 | He, et al. 2019 |
| *Citrus sinensis.* | *CcGA20ox1* | XP_006490288.1 | García-Hurtado, et al., 2012 |
| *Arabidopsis thaliana* | *ATGA20ox1* | NP_194272.1 | Han, et al., 2010 |
|  | *ATGA20ox2* | NP_001332048.1 | Han, et al., 2010 |
|  | *ATGA20ox3* | NP_196337.1 | Han, et al., 2010 |
|  | *ATGA20ox4* | NP_176294.1 | Han, et al., 2010 |
|  | *ATGA20ox5* | NP_175075.1 | Han, et al., 2010 |
| *Solanum lycopersicum* | *SlGA20ox1* | NP_001234070.1 | Irene, et al., 2007 |
|  | *SlGA20ox2* | NP_001234628.2 | Irene, et al., 2007 |
|  | *SlGA20ox3* | NP_001234579.1 | Irene, et al., 2007 |
| *Oryza sativa* | OsGA20ox1 | XP_015628526.1 | Han, et al., 2010 |
|  | OsGA20ox2 | XP_015627721.1 | Han, et al., 2010 |
|  | OsGA20ox3 | XP_025882772.1 | Han, et al., 2010 |

5


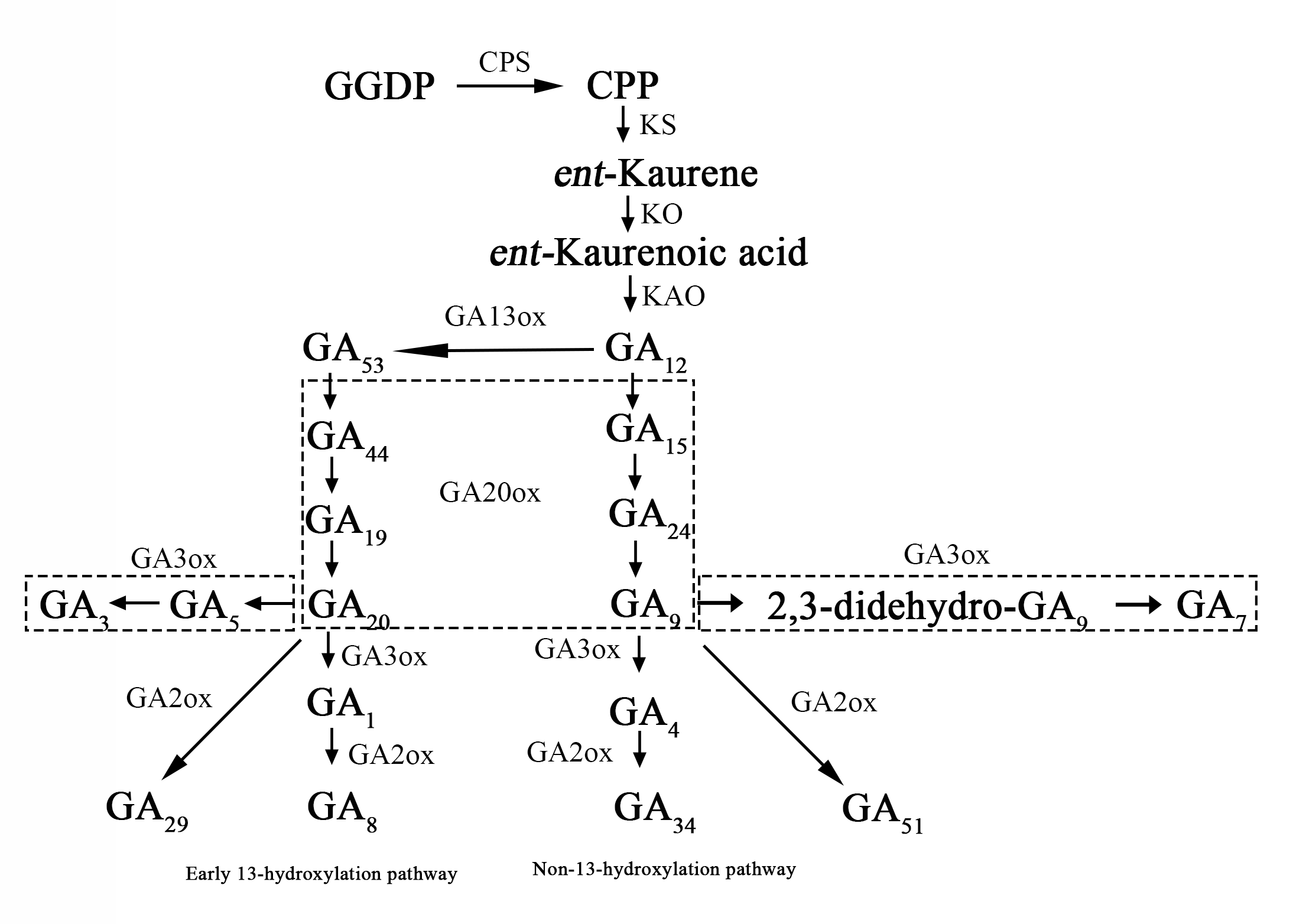


**Supplementary Figure S1 The pathway of gibberellin biosynthesis.** The same enzyme act on the pathway was marked in a black rectangle. CPS, coalydiphosphate synthase; KS, ent-kaurene synthase; KO, ent-kaurene oxidase; KAO, ent-kaurenoic acid oxidase; GA13ox, gibberellin 13-oxidase; GA2ox, gibberellin 2-oxidase.


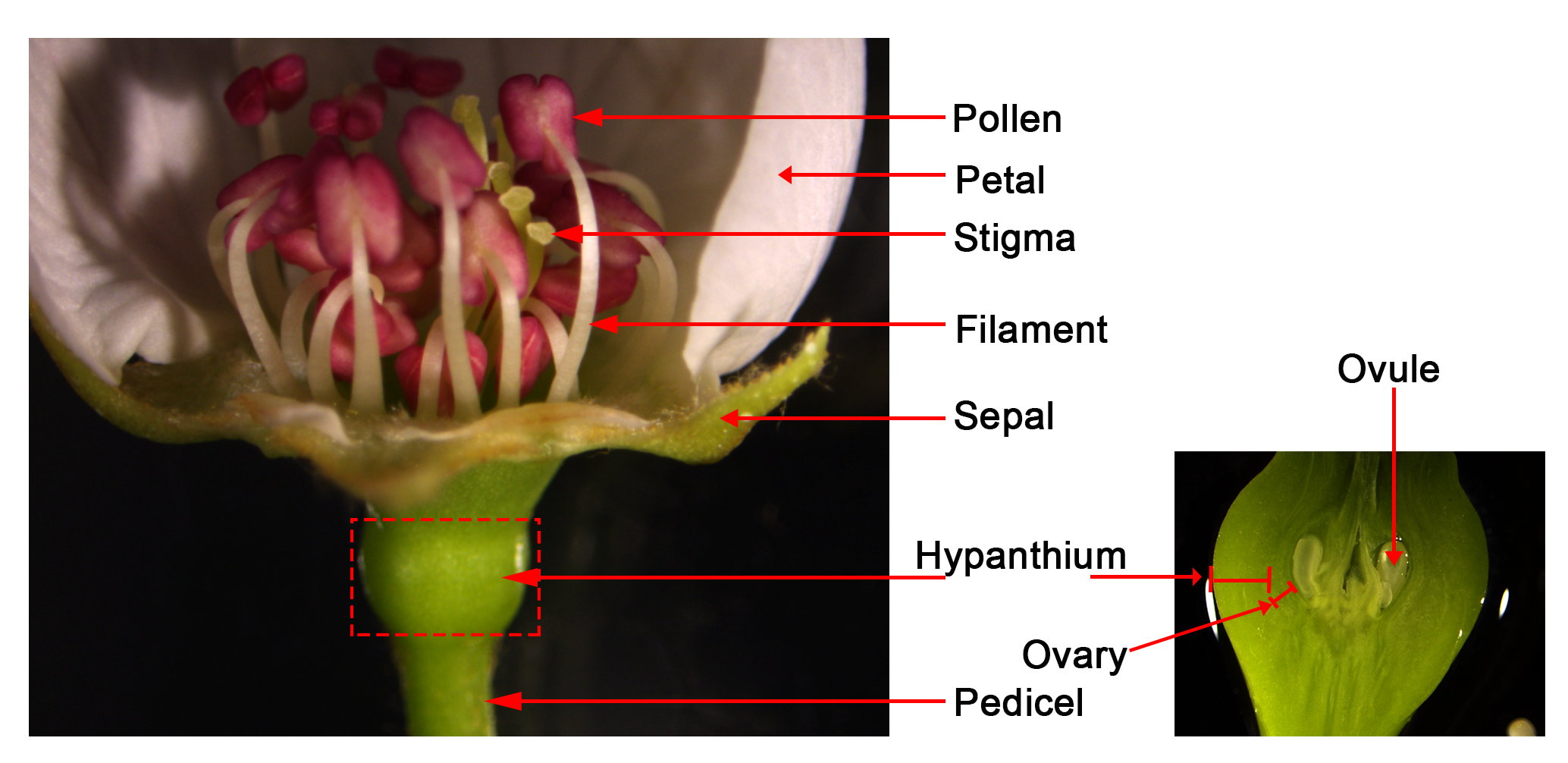


**Supplementary Figure S2** **The information of pear floral organ.** Red arrows refer to different tissues of flower organs. The red dotted rectangle represents the sample of whole fruit, which contains hypanthium, ovary and ovule, for assay in this study.


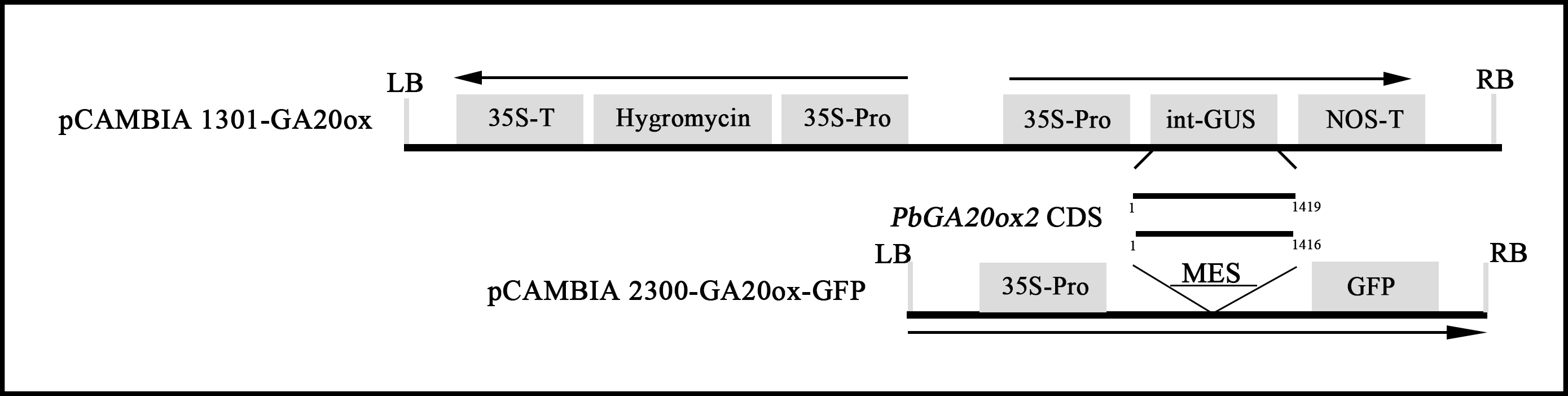


**Supplementary Figure S3 Construction of the recombinant plasmid.** The structural of the recombinant plasmid of pCambia 1301 with *PbGA20ox2* and pCambia 2300-GFP with *PbGA20ox2*. 35S-T, CaMV35S terminator; 35S-pro, CaMV 35S promoter; NOS-T, NOS terminator; int-GUS, GUS reporter gene with a plant intron; LB, left T-DNA border; RB, right T-DNA border; MES, multiple cloning site.


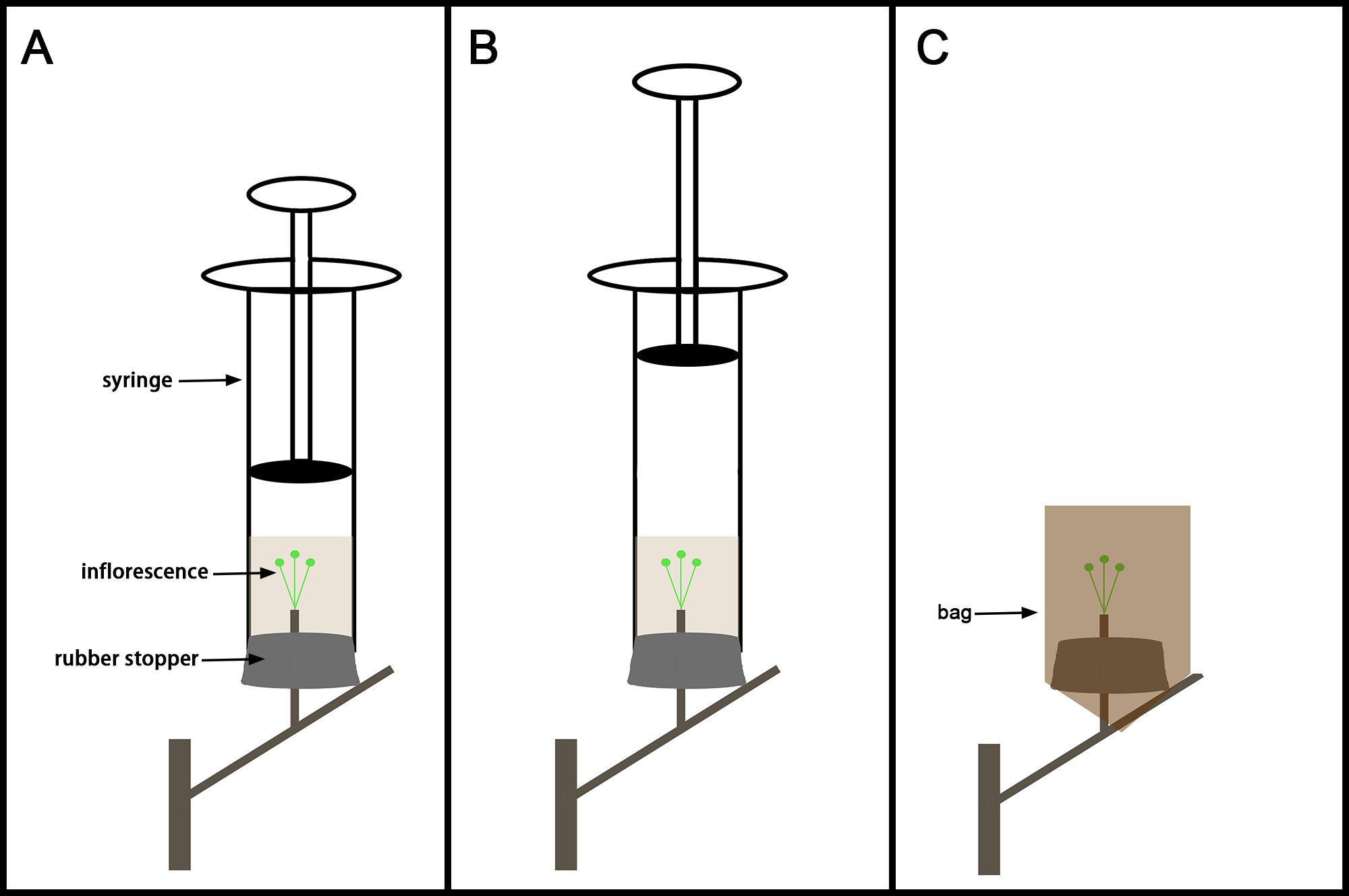


**Supplementary Figure S4 The simplified diagram of transient overexpression assay.** (A) The syringe was fixed on the inflorescence to form a sealed environment, and infection solution was poured into the syringe and soaked the whole inflorescence completely. (B) Pulled the piston to form a negative pressure in the syringe and hold for 10 minutes. (C) Removed the syringe and bagged the inflorescence to prevent pollination.


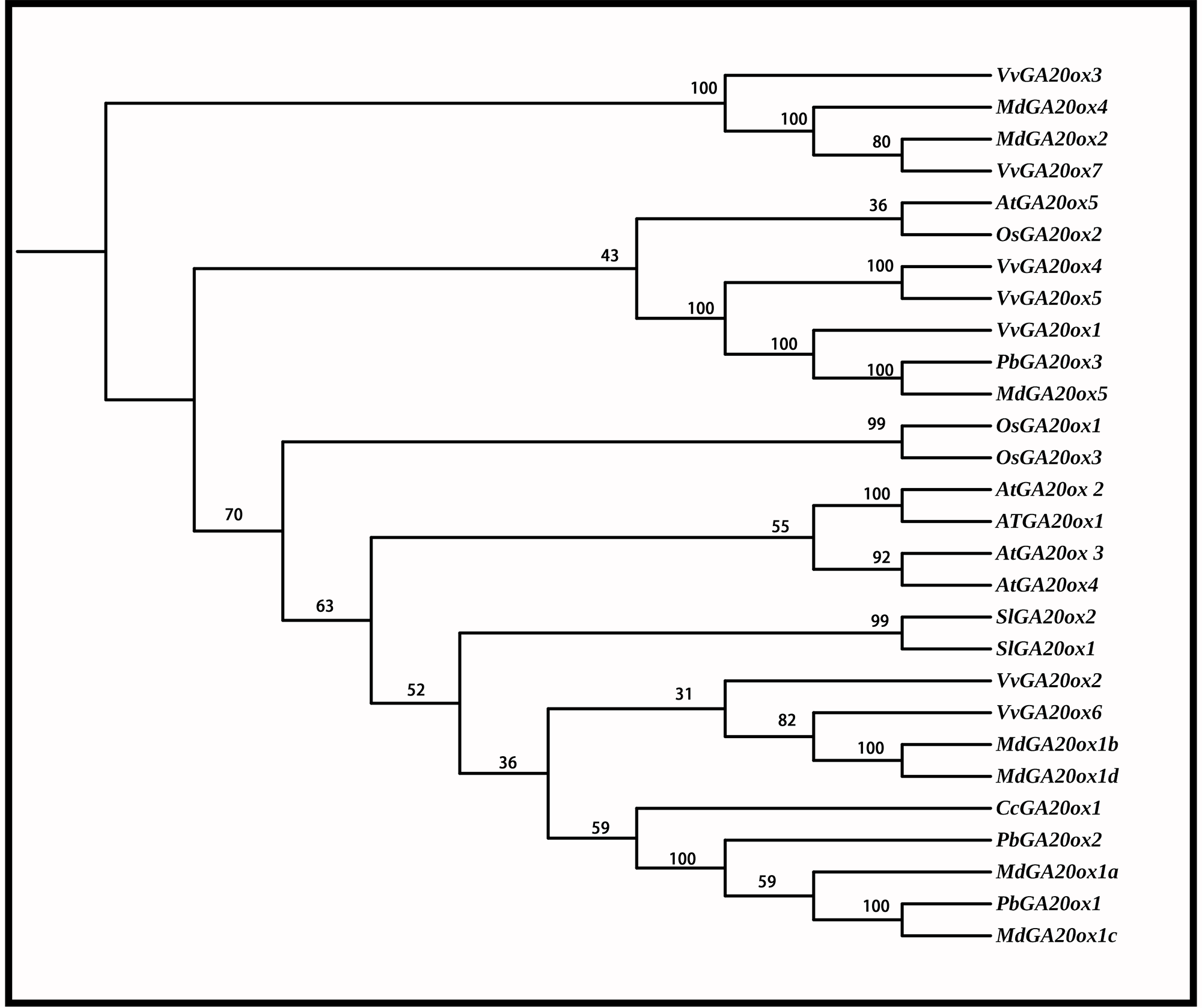


**Supplementary Figure S5 Phylogenetic tree of GA20 oxidase obtained from different plants.** Pb, *Pyrus bretschneideri*; AT, *Arabidopsis thaliana*; Md, *Malus domestica*; Sl, *Solanum lycopersicum*; Os, *Oryza sativa*; Vv, *Vitis vinifera*; Cc, *Citrus sinensis*.


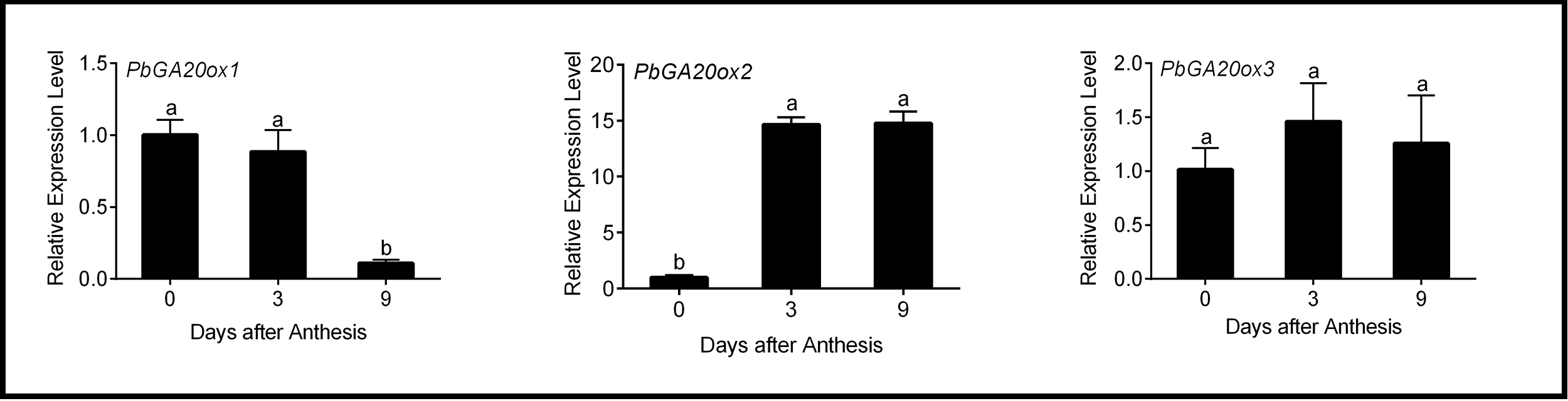


**Supplementary Figure S6 The expression profiles of three *PbGA20ox* genes in ovules.** A qRT-PCR assay was used to determine the relative expression level of *PbGA20ox* genes in ovules. Values shown are means ± SD (n = 3). Significant differences (*P* < 0.05) among different tissues as determined by Duncan’s test were indicated using different lowercase letters.


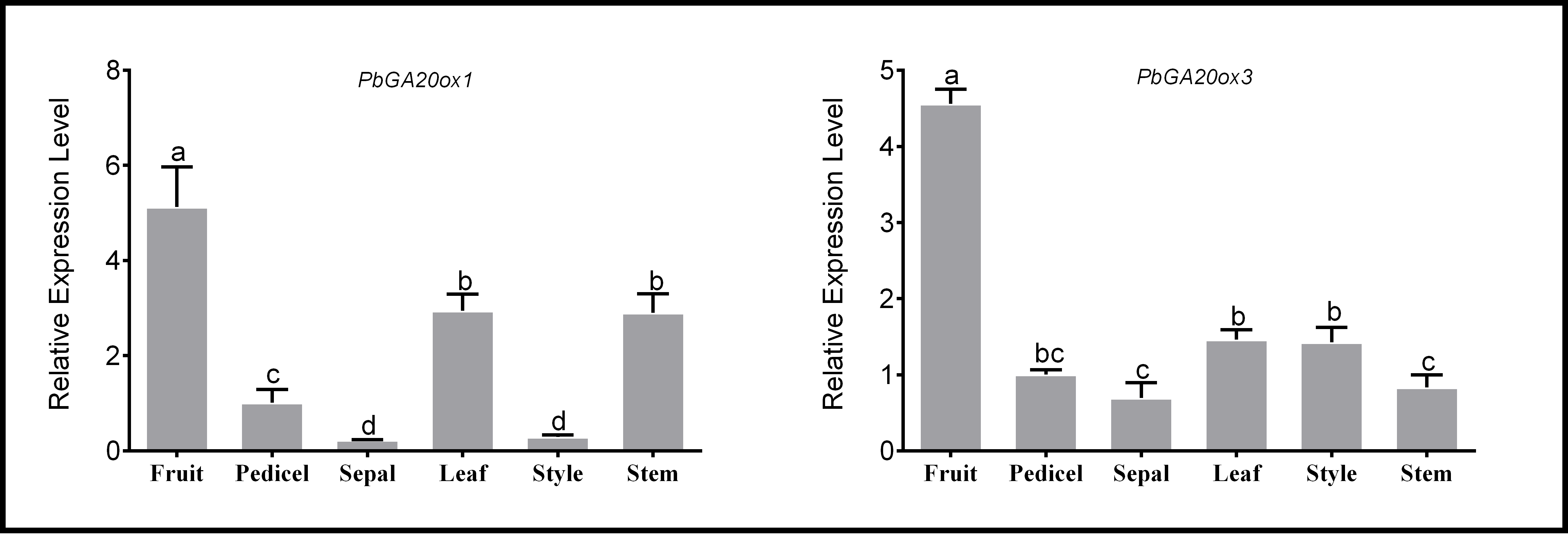


**Supplementary Figure S7 Tissue-specific expression profiles of *PbGA20ox1* and *PbGA20ox3*.** A qRT-PCR analysis was used to determine the relative expression level of *PbGA20ox* in various tissues and organs. Values shown are means ± SD (*n* = 3). Significant differences (*P* < 0.05) among different tissues as determined by Duncan’s test are indicated using different lowercase letters.


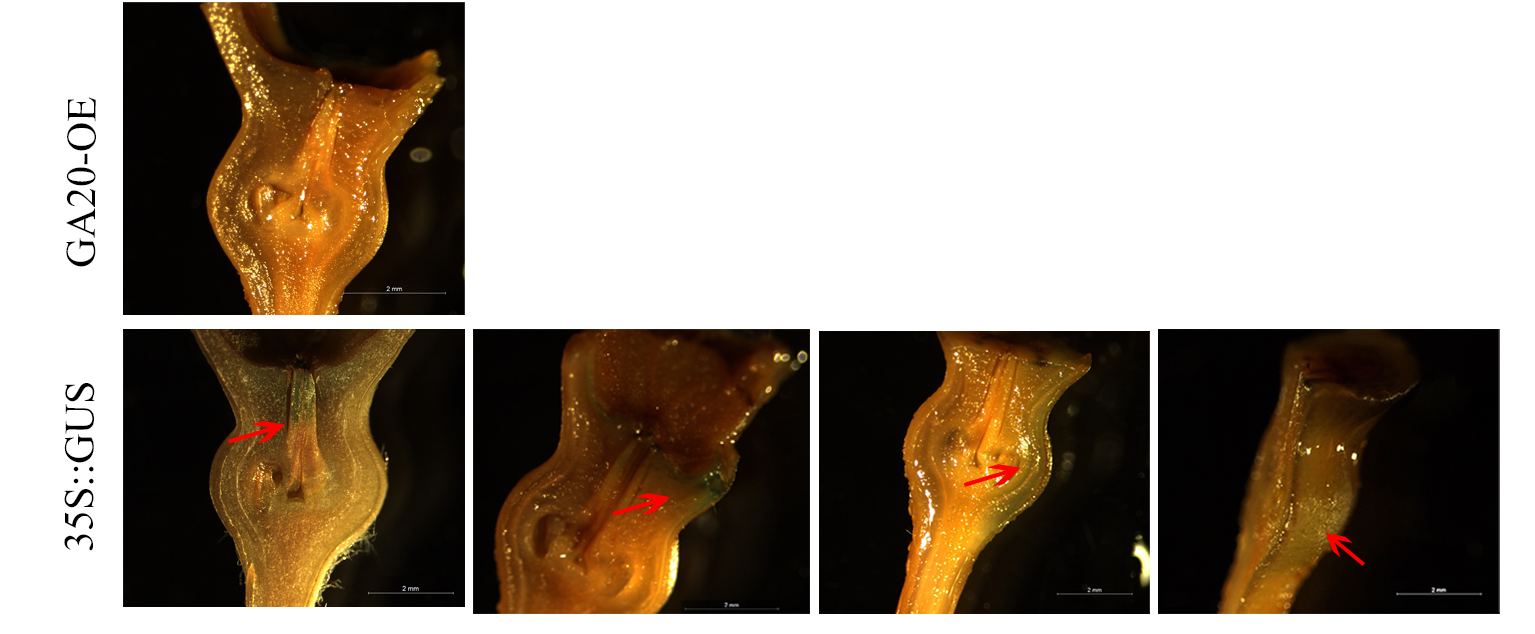


**Supplementary Figure S8 The GUS-staining assay of pear inflorescence.** The GUS-stained ‘Dangshansu’ pear infiltrated by pCambia 1301-GUS (35S:: GUS) and pCambia 1301-GA20ox2 (GA20ox-OE).

**
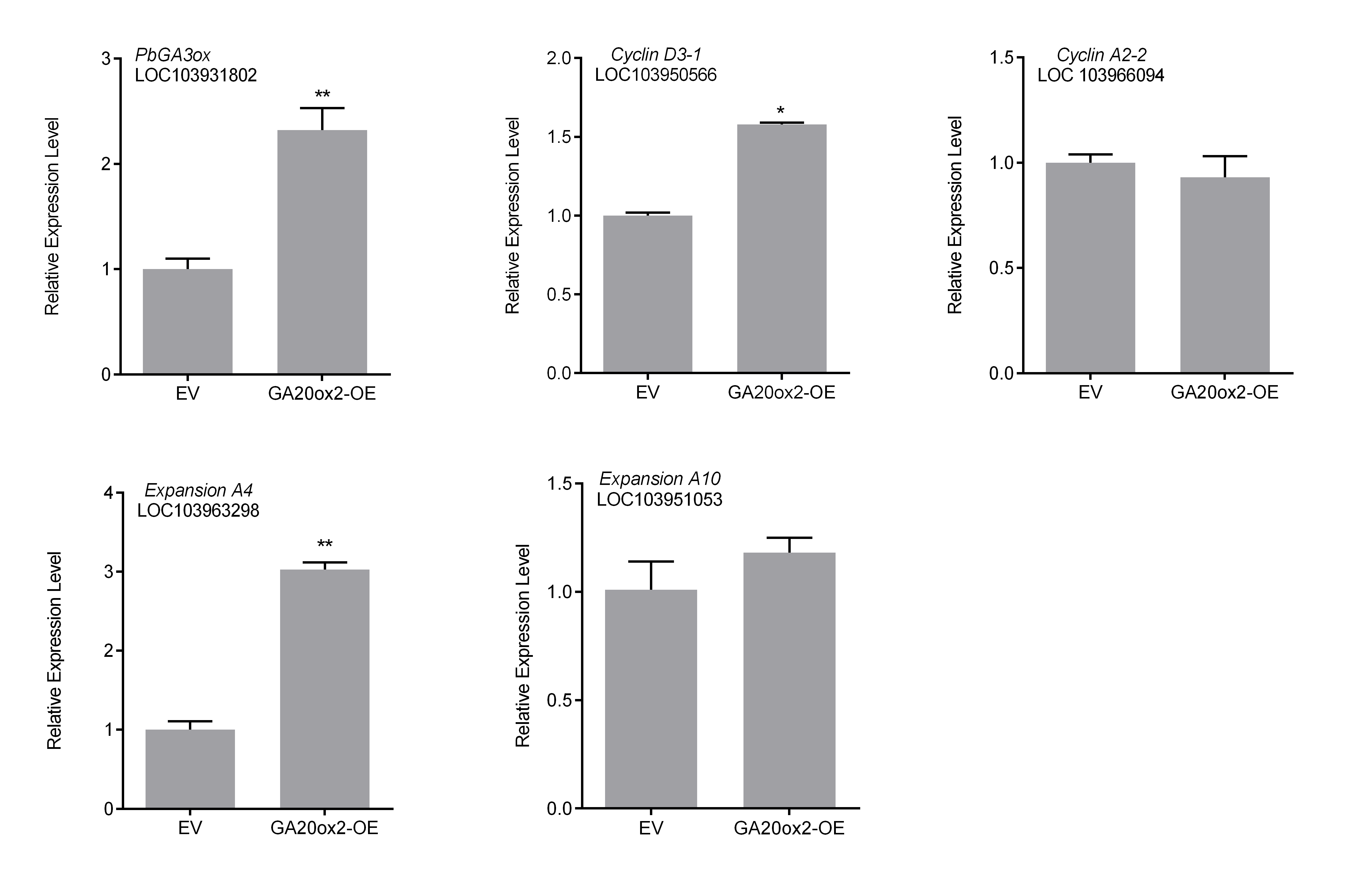
**

**Supplementary Figure S9 The expression profiles of *GA 3-oxidase*, cell division-related and expension-related genes in the overexpressing *PbGA20ox2* fruits.** Asterisks denote a significant difference (**P* < 0.05; Student’s t-test) between control and GA20ox2-OE fruit. EV, empty vector; GA20ox2-OE, *PbGA20ox2* overexpression.

**References**

*Guitton, B., Kelner, J. J., Velasco, R., Gardiner, S. E., Chagne, D., and Costes, E. (2012). Genetic control of biennial bearing in apple. J. Exp. Bot. 63 (1), 131–149. doi: 10.1093/jxb/err261*

*He, H., Liang, G., Lu, S., Wang, P., Liu, T., Ma, Z., et al. (2019). Genome-wide identification and expression analysis of GA2ox, GA3ox, and GA20ox are related to gibberellin oxidase genes in Grape (Vitis vinifera L.). Genes 10 (9),*

*p.680. doi: 10.3390/genes10090680*

*Irene, O., Francesca, S., Riccardo, C., Lorenzo, M., Nello, C., Gian, P. S., et al. (2007). Tomato fruit set driven by pollination or by the parthenocarpic fruit allele are mediated by transcriptionally regulated gibberellin biosynthesis. Planta 226, 877–888. doi: 10.1016/j.plantsci.2010.11.004*
